# Recent Advances and Future Strategies in Chemical Water Shutoff for Gas Reservoirs Under Harsh Conditions

**DOI:** 10.3390/molecules31081281

**Published:** 2026-04-14

**Authors:** Zhenkun Dai, Ming Yue

**Affiliations:** 1 Karamay Municipal Digital Development Bureau, Karamay 834000, China; ddzzkk@sina.cn; 2 School of Resources and Safety Engineering, University of Science and Technology Beijing, Beijing 100083, China

**Keywords:** development of gas reservoirs, advanced chemical plugging agents, selective water control technologies

## Abstract

Water invasion has become a critical challenge during the late-stage development of gas reservoirs, particularly under harsh conditions characterized by high temperature, high salinity, and strong reservoir heterogeneity. Chemical water shutoff technologies have thus gained increasing attention as effective solutions for selectively restricting water production while preserving gas deliverability. This review systematically summarizes recent advances in chemical water shutoff for gas reservoirs, focusing on polymer gels, nanocomposite materials, relative permeability modification agents, and emerging functional fluids. The reviewed materials are analyzed in terms of dominant sealing mechanisms, gas–water selectivity, reservoir adaptability, and performance under extreme formation conditions. By critically comparing their advantages, limitations, and field applicability, key challenges related to deep placement, selective sealing, long-term stability, and engineering controllability are identified. To address these limitations, emerging concepts such as zonal synergistic water control and bioinspired gas–water barriers are discussed, integrating wettability regulation, multiscale sealing, and adaptive material responses. These strategies provide a conceptual framework and research direction for the design of next-generation, efficient, and sustainable chemical water shutoff systems in complex gas reservoirs.

## 1. Introduction

Water invasion is a critical issue that undermines the production stability and economic performance of gas wells during gas reservoir development. It refers to the process by which water from adjacent aquifers migrates into the gas-bearing formations through seepage channels, influenced by pressure gradients, fractures, and pore structures [1,2]. With increasing production intensity, the bottomhole pressure drops rapidly, promoting the formation of tongue-like, finger-like, or bottom-water wedge-shaped intrusions that significantly reduce gas output and exacerbate problems such as equipment corrosion, sand production, and wellbore blockage [3]. The mechanism of water invasion is highly complex, influenced by pore structure, flow characteristics, wettability, aquifer thickness, and unsteady multiphase flow. In pore-type reservoirs, bottom-water breakthrough is common, whereas in fractured or fracture–pore composite reservoirs, natural and hydraulic fractures serve as rapid conduits, making water invasion more abrupt and difficult to predict [4,5]. Furthermore, gas–water interactions under multiphase flow conditions lead to irregular, fluctuating interfaces, complicating prediction and control [6].

Macroscopically, water invasion alters reservoir pressure distribution and flow systems, leading to increased water saturation, reduced effective pore volume, and a decline in gas relative permeability. Persistent water coverage creates immobile zones that impair productivity and shorten reservoir economic life [7,8]. Additionally, continued water encroachment at the gas–water interface can induce local stress changes, causing wellbore collapse and increasing operational and environmental risks [9].

In practice, control strategies for water invasion are generally classified into two main categories: physical and chemical. Physical methods (e.g., mechanical packers, drainage systems, and water-tight tubing) offer short-term control but are limited by geologic complexity and placement accuracy [10,11]. In contrast, chemical water shutoff technologies—by injecting specific agents to form physical or chemical barriers in high-permeability water-bearing zones—modify flow paths and selectively block water while maintaining gas flow. This makes them an increasingly important focus of research [12,13].

Typical agents include polymer gels, microemulsions, and particulate or colloidal materials [13,14,15,16], which can form impermeable structures in pores or fractures with minimal gas flow impairment. However, in high-temperature, high-pressure, and high-salinity environments, challenges remain, including precipitation or degradation of agents, poor placement in heterogeneous fractures, and limited long-term barrier stability [12,16,17,18]. These issues highlight the need for advances in molecular design, rheology control, and injection optimization, all of which should be supported by field trials.

Effective material development requires understanding reservoir geology and invasion characteristics, including strong heterogeneity, tight matrix (k < 1 mD), zones of extreme permeability contrast, high pressure (>10 MPa), elevated temperature (120–180 °C), and complex flow regimes (bottom/edge water and condensate interactions) [19,20,21]. This review proceeds to comprehensively analyze material selection, reservoir applicability, water-blocking performance, and technological direction, covering polymer–microsphere matching, surfactant-induced wettability alteration, nanocomposite enhancement, and metrics such as cost, injectability, and environmental compatibility.

Future directions include multimaterial hybrid systems, full-scale plugging platforms, CO_2_-assisted processes, and integration of real-time monitoring and numerical modeling to establish closed-loop design–injection–evaluation workflows. In summary, water invasion remains a key technical challenge with significant economic implications in gas production. Its effective control relies on interdisciplinary collaboration and advanced technologies, which together will enable the efficient and sustainable development of gas resources.

Previous reviews have mainly focused on oil reservoirs, single material categories, or field case summaries, with limited attention to gas reservoir-specific challenges such as high-temperature and high-salinity environments, fracture–matrix heterogeneity, and gas–water selectivity.

This review focuses on chemical water shutoff technologies specifically for gas reservoirs. The literature is analyzed based on (i) material categories, (ii) dominant sealing mechanisms, and (iii) reservoir applicability under harsh conditions. By integrating material performance with gas–water flow mechanisms, this review aims to identify critical knowledge gaps and emerging strategies for future development.

### 1.1. Scope and Literature Search Strategy

To improve transparency and reproducibility, this review followed a structured literature search and screening workflow. We focused on chemical water shutoff technologies tailored to gas reservoirs under harsh conditions (high temperature and salinity, fracture–matrix heterogeneity, and gas–water selectivity).

Databases and search period: Web of Science Core Collection, Scopus, and OnePetro were queried for studies published between January 2000 and December 2024. Supplementary searches were performed in Google Scholar for key terms and in the reference lists of relevant reviews and field reports.

Search keywords (examples): (“gas reservoir” OR “gas well” OR “condensate gas”) AND (“water shutoff” OR “conformance control” OR “relative permeability modification” OR “wettability alteration” OR “gel” OR “microgel” OR “foam” OR “nanofluid” OR “microemulsion”).

Inclusion criteria: (i) gas reservoir or gas well water control; (ii) chemical agent forms an in situ or placed barrier with reported performance (lab coreflood, microfluidics, or field case); (iii) the study reports at least one of the following: plugging strength/differential pressure, permeability reduction, selectivity (gas vs. water), injectivity/migration, or stability under temperature/salinity.

Exclusion criteria: (i) purely mechanical isolation without chemistry; (ii) studies focused solely on oil reservoirs without transferable gas–water selectivity insights; (iii) insufficient methodological details to interpret performance; (iv) duplicate publications of the same dataset (the most complete version was retained).

### 1.2. Data Extraction and Synthesis

For each included study, we extracted reservoir conditions (temperature, salinity, permeability/porosity, fracture presence), agent composition and form (gel, particle, foam, nanocomposite, RPMA), key performance metrics (water-phase permeability reduction, gas-phase impairment, differential pressure/plugging strength, placement radius, and longevity), and implementation parameters (preflush, injection sequence, isolation tools). Evidence was synthesized by material category and by dominant mechanism (precipitation/curing, crosslinking network formation, particle bridging/swelling, interfacial wettability regulation, and nano-reinforcement). Cross-category comparisons are summarized in Section 4, and the proposed decision framework is described in Section 5.

## 2. Water Shutoff in Gas Reservoirs: Research and Application

With the continued development of natural gas resources, water invasion has emerged as a critical challenge limiting the stable production and economic performance of gas wells. Compared to oil reservoirs, gas reservoirs often exhibit lower permeability, more complex pore structures, and highly developed natural fractures, making water intrusion more abrupt and less predictable [22,23,24]. Traditional physical isolation methods are often insufficient for deep and effective water control. In this context, chemical water shutoff technologies characterized by their tunability, selectivity, and adaptability have gained increasing attention and application in recent years [25]. Significant progress has been made globally in terms of agent types, mechanisms of action, laboratory evaluation methods, and field deployment strategies. However, systematic assessments of their applicability under gas reservoir-specific conditions and large-scale field experience remain limited, as summarized in Table 1. Therefore, a comprehensive review of current advances and field practices in chemical water shutoff is essential for promoting the engineering implementation of high-performance agents and optimizing water control strategies for complex gas reservoirs.

### 2.1. Inorganic Salts and Resin-Based Water Shutoff Agents

Particle-based plugging agents play a critical role in water shutoff operations by physically obstructing high-permeability channels through filtration, bridging, or expansion-driven blocking effects. Upon injection, these particles typically undergo a sequence of dispersion, pore entry, and in situ accumulation. When the particle size is sufficiently large—typically exceeding one-third of the pore throat diameter—a tight filter cake can form at pore constrictions, leading to effective channel occlusion and redirection of water flow [25,28]. Materials employed in this category span a broad spectrum, including inorganic solids such as clays, fly ash, and mineral powders, as well as polymeric microspheres and flexible rubber granules. These agents exhibit strong mechanical resistance (≈10 MPa) and tolerate harsh reservoir conditions ranging from 25 to 200 °C and high salinity levels [26]. However, gravitational settling, particle aggregation in dominant flow paths, and limited adaptability to heterogeneous formations often compromise their deep migration and coverage efficiency [29]. In addition, mechanical degradation under stress can reduce long-term sealing performance. Expandable microgels or preformed particles further enhance plugging depth by leveraging water-triggered swelling. These materials can initially pass through pore networks in a compact state, then expand upon hydration to temporarily seal pathways. Under elevated pressure gradients, they may deform or rupture, allowing fragments to advance deeper into the formation and reassemble at narrower sites [30,31]. Their performance is closely associated with swelling kinetics, viscoelastic response, injection concentration, and flow regime.

From an operational perspective, particle-based systems are favored for their relatively low cost, field applicability, and tunability through particle size gradation [27]. Recent studies have focused on optimizing multiscale particle blending strategies to improve conformance control [32]. For example, staged injection of coarse-to-fine particle systems extends the effective plugging radius. Advanced visualization techniques (e.g., micro-CT, fluorescence imaging) have enabled researchers to observe particle transport and retention behaviors in complex pore–fracture systems, whereas numerical models coupling flow dynamics with particle mechanics are being developed to predict sealing patterns under varying injection scenarios.

Despite progress, several limitations persist. Particle agents tend to accumulate in dominant flow channels, leaving low-permeability zones undertreated [31,33]. High-temperature and saline conditions may cause dehydration or aggregation of polymer-based materials. Moreover, the irreversibility and stability of plugging layers remain open concerns, especially for long-term reservoir control.

### 2.2. Polymer-Based Water Shutoff Agents

Polymer-based water shutoff agents are among the most widely applied chemical materials for water control in gas reservoirs [25,31,34]. Leveraging their high viscoelasticity and tunable crosslinking behavior, these agents form high-strength gels or viscous barriers within high-permeability channels to effectively block water flow while preserving gas conductivity. Their action modes are broadly classified into three types: (1) adsorptive retention of linear polymers, where molecular entanglement and shear thickening increase water-phase resistance; (2) physically crosslinked weak gels for flexible control under low-temperature or slow-flow conditions; and (3) chemically crosslinked three-dimensional gel networks, suitable for long-term sealing of deep, high-water-cut zones. Common polymers include polyacrylamide (PAM), modified starches, xanthan gum, and various amphoteric copolymers. These are typically combined with crosslinkers such as Cr^3+^ or organic agents to adjust gelation time, strength, and thermal/salt resistance [30,31]. To address the hydrolysis and degradation issues in high-temperature and high-salinity reservoirs, recent research has developed thermally stable polymer backbones and self-healing dynamic crosslinking networks, significantly improving durability and reservoir compatibility [35,36,37].

Laboratory and field data have shown that polymer gels can maintain shear strengths above 5 MPa and stable sealing for several months under conditions of 120–150 °C and salinity up to 200,000 mg/L [38,39]. Some systems exhibit regelation ability after multiple stimulation or washing cycles, offering potential for reversible control.

Key challenges remain: (1) channeling in fractured formations, leading to reduced plugging efficiency; (2) limited formulation stability prior to injection, resulting in narrow operational windows; and (3) insufficient deep migration, especially in ultra-low-permeability reservoirs. Future developments should focus on hybrid systems combining polymers with microspheres or surfactants, as well as real-time monitoring and numerical simulation to optimize injection strategies and improve water-blocking selectivity and long-term performance in complex reservoirs.

### 2.3. Foam-Based Water Shutoff

Foam systems have emerged as a versatile solution for selective water shutoff, especially in fractured and heterogeneous gas reservoirs [40,41]. By generating a dispersed gas–liquid structure within the porous medium, foam effectively reduces water phase mobility while maintaining or restoring gas flow capacity. This selectivity arises from several interrelated mechanisms [39,42]. First, foam introduces enhanced capillary resistance at pore throats due to the small radius of curvature of the gas–liquid interfaces. The resulting capillary pressure acts as a dynamic barrier that prevents further water penetration into high-permeability zones. Second, the viscoelastic nature of foam introduces significant flow resistance. Foam bubbles undergo continuous deformation, coalescence, and rupture, which dissipate energy and hinder water movement. Third, due to differences in wettability, foam preferentially accumulates in water-wet channels while being less stable in gas-dominated zones, thereby facilitating selective phase control [43,44,45]. Finally, foam systems can be engineered to remain stable over time or to degrade controllably, depending on reservoir demands.

Laboratory studies have shown that foam behavior is highly sensitive to surfactant type, gas fraction (foam quality), temperature, salinity, and pore geometry. For example, nonionic and zwitterionic surfactants often perform better in high-salinity reservoirs, maintaining foam stability under challenging chemical environments [46]. Microfluidic and coreflooding experiments have demonstrated foam’s ability to form stable “foam banks” in pore networks or narrow fractures, improving water-blocking efficiency with minimal damage to gas conductivity [47,48].

Despite these advantages, foam-based shutoff technologies face several practical limitations. Foam stability deteriorates significantly in ultra-high-temperature (>120 °C) or ultra-high-salinity (>100,000 mg/L) environments [43]. Additionally, reservoir heterogeneity can lead to non-uniform foam propagation, causing issues such as channeling, bypassing, or erratic mobility. Moreover, once foam is destabilized, reestablishing its original blocking structure is challenging, raising concerns about long-term effectiveness and retreatability [43].

### 2.4. Wettability Reversal Agents (WRAs)

Wettability reversal agents (WRAs) are a class of functional chemicals designed to selectively block water flow in gas reservoirs by altering the surface wettability of reservoir rocks [49]. Their core function lies in transforming hydrophilic or neutral mineral surfaces into gas-wet (or oil-wet) states, thereby suppressing water-phase migration in pores and fractures while enhancing gas-phase permeability. This enables efficient water shutoff with minimal impact on gas deliverability [50,51]. At the microscopic level, WRAs adsorb onto rock surfaces via electrostatic attraction, van der Waals forces, or hydrophobic interactions, forming a dense and stable interfacial layer. This adsorbed film displaces pre-existing water films and reduces the rock’s affinity for water, leading to a significant increase in contact angle and a decrease in water-phase mobility. Additionally, WRAs can reduce rock–water interfacial tension and promote gas-phase spreading. Some formulations exhibit pH or ion-sensitive behaviors, allowing targeted release and spatial control under specific formation conditions.

Laboratory studies using contact angle measurement, NMR analysis, and microfluidic visualization have demonstrated that various WRAs, including cationic, nonionic, and nanoemulsion-based systems, effectively alter wettability across multiple rock types (e.g., sandstones, carbonates) [52,53]. Cationic surfactants tend to offer more stable adsorption in low-salinity environments, whereas nanoemulsion carriers enable robust performance under high-temperature (>120 °C) and high-salinity (>150,000 mg/L) conditions. In some cases, contact angle increases of over 30° and reductions in water-phase permeability exceeding 50% have been reported. Microscale visualization and pore network simulations further reveal the preferential adsorption of WRAs in high-permeability zones, followed by diffusion into lower-permeability regions, forming multiscale wettability barriers. In field applications, WRAs are often used in conjunction with other chemical agents, such as foams, gels, or polymer microspheres, to establish staged water control [54]. A common strategy is to precondition the reservoir with a WRA to modify the rock surface before injecting plugging agents, thereby enhancing long-term effectiveness. In fractured formations, horizontal wells, and condensate gas reservoirs with active edge or bottom water, WRA-assisted treatments have demonstrated significant improvements. Field cases have reported up to 60% reductions in water production and sustained shutoff performance for over two years using zonal injection and pH- or temperature-triggered WRA systems.

Nevertheless, several technical challenges remain. These include ensuring long-term adsorption stability in high-temperature and high-salinity formations, achieving uniform action in geologically heterogeneous reservoirs, and maintaining chemical compatibility during co-injection with other agents. Future research should focus on the design of multifunctional and responsive surfactants, development of intelligent WRA systems, and optimization of injection schemes [55]. Integrating online monitoring technologies and tracers will also be critical for tracking wettability transitions in real time and enabling adaptive control.

Wettability reversal agents alter rock surfaces from water-wet to gas-wet conditions, thereby significantly reducing water-phase permeability while maintaining gas flow pathways for selective water control [55,56]. These materials form a hydrophobic adsorption layer on the rock surface, increasing the contact angle, disrupting capillary water films, and suppressing water spreading. Common WRAs include cationic and nonionic surfactants as well as nanoemulsion systems. Experiments have shown that these agents maintain good stability under high-temperature and high-salinity conditions, achieving reductions in water-phase permeability exceeding 50%. Microscopic visualization and numerical simulations confirm their preferential adsorption in high-permeability flow channels. In field applications, WRAs are often used in combination with gels or foams, making them suitable for fractured formations and condensate gas reservoirs [57]. The main challenges lie in ensuring long-term adsorption stability under harsh conditions and achieving uniform action in heterogeneous formations. Given their excellent injectivity, low formation damage, and strong selectivity, WRAs have become an important emerging technology for chemical water shutoff in gas reservoirs.

As gas reservoir development enters the middle to late stages, water invasion has become an increasingly serious issue, driving a growing demand for chemical water shutoff technologies in field operations. In recent years, field trials of various shutoff agents have been conducted in typical gas reservoirs both domestically and internationally, covering a wide range of reservoir types, including fractured, tight, and condensate gas reservoirs as summarized in Table 2 [58]. These efforts have gradually advanced engineering experience in agent selection, injection parameter optimization, and performance evaluation. In North America, companies such as Shell and Chevron have widely applied polymer gels, microspheres, and foam-based composite systems in shale gas and tight sandstone reservoirs to selectively block water invasion along horizontal well sections [59]. In China, chemical water shutoff field applications have also been performed in regions such as the Kuqa foreland fault belt of the Tarim Basin and the Sulige gas field in the Ordos Basin. Field practices show that the effectiveness of water shutoff treatments is influenced by multiple factors, particularly reservoir heterogeneity, water invasion mechanisms, and injection strategies [60].

## 3. Advanced Chemical Water Shutoff Materials for Gas Reservoirs

### 3.1. Polymeric Materials and Gel Systems for Water Shutoff

#### 3.1.1. Field Applications of Polymer-Based Water Shutoff

In the 1970s and 1980s, early water shutoff efforts in gas wells primarily relied on single-component polymers, with acrylamide-based systems being the most commonly used [61]. One of the earliest field trials was conducted in 1977 at a gas field in Colorado, USA, using a PAM solution. However, the treatment was unsuccessful, and the well quickly ceased production afterward [62,63]. Subsequent efforts in France’s Verena gas reservoir involved the use of copolymers composed of acrylamide and acrylate monomers. These copolymers exhibited responsive behavior: contracting in high-salinity water and expanding in low-salinity conditions. This behavior allowed for adjustments in relative permeability between gas and water phases, enabling a degree of disproportionate permeability reduction (DPR) [63]. Despite initial promise, long-term production improvement remained limited, as shown in Table 3. To address the thermal and salinity constraints of traditional HPAM and PAM systems, researchers began incorporating stabilizing additives and developing ternary or modified copolymers. For example, activators can function as stabilizers or form coordination complexes with polymer chains, enhancing thermal resistance [64]. A notable example is the Wales gas well in Canada, where PAM-based treatment successfully mitigated water coning issues [64,65].

Ternary copolymers with interwoven molecular networks formed from monomers such as acrylamide (AM), vinyl sulfonate (VS), and vinyl acetate (VA) have shown superior structural integrity and stability in harsh reservoir environments. In a northern German gas reservoir, such a ternary system reduced water production from 90 m^3^/d to less than 1 m^3^/d while restoring gas output to greater than 105 m^3^/d [61]. In western Germany, the use of a low-molecular-weight, partially sulfonated acrylamide-based ternary copolymer led to a fivefold increase in the gas–water ratio (GWR), demonstrating its effectiveness under high-salinity conditions [66]. These historical applications highlight the evolution of polymer water shutoff materials from simple acrylamide formulations toward more robust, chemically engineered copolymers capable of performing under extreme reservoir conditions.

In high-temperature and high-salinity gas reservoirs, a combined treatment strategy using a preflush solution followed by a modified polymer system has proven more effective for water shutoff. Conventional polymer-based agents, such as PAM and HPAM, often exhibit high viscosity and elevated resistance factors, which can unintentionally hinder both gas and water flow, especially in low-permeability or low-productivity formations [67]. This can lead to complete blockage of flow channels and loss of deliverability. To overcome these limitations, ternary copolymers and their modified variants have gained attention given their lower molecular weight and reduced viscosity. These systems can navigate narrow pore structures more effectively and exhibit improved injectivity under tight formation conditions. Their balanced rheological behavior allows for sufficient placement depth without causing excessive flow resistance, making them better suited for delicate gas–water systems where maintaining gas mobility is critical. By tailoring the polymer structure and optimizing injection sequences, these advanced formulations offer a promising solution for enhancing water control while minimizing formation damage, particularly in challenging reservoirs with complex mineralogy and ultra-low permeability [68].

#### 3.1.2. Field Applications of Polymer Gel-Based Water Shutoff

During gelation, polyacrylamide-based polymers form three-dimensional networks through crosslinking with either organic or inorganic agents. In organic systems, covalent bonding occurs between amide groups and the crosslinker, enhancing the rigidity of polymer chains and increasing the number of effective crosslinking sites [69]. In contrast, inorganic crosslinkers (e.g., metal ions) often initially induce intramolecular folding into single-chain microgels, which subsequently aggregate through intermolecular linkages to form larger network structures, resulting in improved spatial stability [69]. Compared with conventional polymer solutions, polymer gels offer superior thermal and salinity resistance, making them more suitable for controlling water production in high-temperature, high-salinity gas reservoirs. Field trials have validated their effectiveness in diverse geological settings, including the Peciko gas field in East Kalimantan (Indonesia) [70], East High Island 285 in the Gulf of Mexico [71], Tainan gas field in Qinghai, China [72], the Bassein gas field in India [73], and a high-temperature well in Oklahoma, USA [73] (see Table 4). In addition to chemical stability, polymer gels exhibit excellent viscoelasticity and resistance to flow-back erosion. For instance, in fractured gas reservoirs, the Sukunka field in British Columbia employed chromium-based gels to treat excessive water production, resulting in a ~75% reduction in the water–gas ratio (WGR) and a 50% increase in gas output [74].

However, high-temperature conditions pose specific challenges to gel systems. Bach et al. [64] observed that gels crosslinked with inorganic agents such as Cr(III) tend to undergo hydrolysis and precipitation at elevated temperatures. These precipitates often fail to migrate effectively to the leading edge of the treatment zone, thereby reducing the depth of penetration and weakening the desired disproportionate permeability reduction (DPR) effect. Therefore, organically crosslinked gel systems are generally preferred in high-temperature applications due to their enhanced thermal stability and deeper placement capabilities.

#### 3.1.3. Adaptability of Polymeric Materials and Gel Systems in Gas Reservoirs

Overall, polymer and polymer gel systems are best suited for reservoirs with moderate pore sizes and medium to high permeability. In such formations, polymers offer good injectivity and continuous plugging capability, enabling effective water shutoff without significantly impairing gas flow. However, due to the relatively weak intermolecular forces between linear polymer chains, conventional systems often suffer from poor thermal and salinity resistance, limited shear stability, and low plugging strength. These limitations make them unsuitable for high-stress environments such as fractured formations or ultra-high-temperature reservoirs. To enhance performance and expand their applicability, two main optimization strategies have been adopted. First, in high-temperature reservoirs, preflush treatments using cold water or activators can help regulate local temperature and hydration conditions, improving polymer placement and crosslinking efficiency. Second, incorporating chemical crosslinkers to form gels or using modified polymer structures can significantly improve the material’s resistance to heat, salinity, pressure, and erosion, thereby making them more effective in fractured, high-salinity, or high-temperature gas wells.

Nevertheless, in reservoirs with extremely high water saturation, the absorbed water in gels can promote shear thinning, reducing residual gas saturation and weakening the effectiveness of disproportionate permeability reduction (DPR) [61]. To address this, advanced crosslinking techniques have been proposed to avoid “dual-phase blockage” of both gas and water. These techniques include generating in situ gas channels through acid-releasing reactions, embedding weakly crosslinked pathways, or inducing external gas-flow conduits during gelation [14]. Such approaches aim to retain selective gas permeability within the plugged zone, enabling more targeted and efficient water control in complex reservoir environments.

### 3.2. Relative Permeability Modification Agents (RPMAs) for Water Control

Relative permeability modification agents are specialized materials designed to selectively reduce water-phase permeability while maintaining relatively unimpeded gas-phase flow, thereby achieving targeted water shutoff in gas reservoirs. These agents are generally classified into two main categories. The first category comprises water-soluble polymer-based systems, which feature lower viscosity, superior injectivity, and better mobility than conventional polymer or gel-type blocking agents. As such, they are often regarded as a more advanced evolution of traditional polymer-based water shutoff technologies [75,76]. The second category includes fluorinated systems, primarily composed of fluorocarbon surfactants. These agents utilize strong interfacial activity and wettability alteration mechanisms to modify multiphase flow characteristics and enhance water–gas selectivity. Both types of agents are gaining increasing attention in the context of fractured, high-salinity, or ultra-low-permeability gas reservoirs, where conventional blocking methods often fall short.

#### 3.2.1. Field Applications of Relative Permeability Modification Agents

Water-soluble polymers are often studied and applied independently as a type of relative permeability modification agent (RPMA). Many researchers consider certain linear polymers or weak polymer gels to function as RPMAs. For example, Chen et al. [22] evaluated HPAM-1 and HPAM-2 as RPMAs, leveraging their ability to form smooth, hydrophilic adsorption layers along pore walls to suppress water-phase mobility while maintaining gas flow—a strategy often described as “blocking water while allowing gas.” Pietrak et al. [77,78] reported a sulfonated acrylamide-based RPMA system that effectively reduced the effective diameter of water flow paths, enhanced water-phase resistance, and demonstrated thermal stability up to 98 °C. Campbell et al. [79] developed a hydrophilic terpolymer (STP) capable of selectively controlling water in high-temperature (>149 °C), high-salinity, and high-velocity gas-bearing sandstone formations. This system demonstrated strong gas–water selectivity and robust field performance. Eoff et al. [80] further advanced this concept by synthesizing a brush-shaped polymer with a methoxy polyethylene glycol (MPEG) backbone that maintained excellent stability under highly saline conditions and was successfully applied in both sandstone and carbonate gas reservoirs.

In contrast, fluorinated RPM systems function primarily by altering rock wettability from liquid-wet to gas-wet states, thus enhancing water-blocking selectivity via a disproportionate permeability reduction (DPR) mechanism. Jin et al. [81] modified nanosilica particles with fluorinated surfactant FG40 to induce a wettability shift toward a gas-wet state, increasing water-phase resistance and improving the productivity of condensate gas reservoirs. Liu et al. [26] developed a fluorocarbon surfactant-based system (WA12) that exhibited excellent thermal stability (up to 170 °C), high chemical resistance, strong hydrophobicity, and salt tolerance up to 70,000 mg/L. Similarly, Wang et al. [82] demonstrated that FG24, a fluorinated surfactant, could convert the rock surface to a gas-wet state, resulting in a greater than 25% increase in both gas relative permeability and gas production rate, while maintaining thermal stability at temperatures up to 120 °C.

Water-soluble polymer-based relative permeability modification agents (RPMAs) have demonstrated moderate thermal stability and are primarily applied in gas reservoirs characterized by low porosity and medium permeability. Notable field trials include applications in Block W of the Lunyu gas reservoir [83], a well in Canada [84], and the Grand Island gas field in Nebraska, USA [84]. In each case, a significant reduction in water production was observed post-treatment, effectively reestablishing gas flow pathways and enhancing overall gas deliverability. Given the strong C–F bonds in their molecular structure, fluorinated surfactant-based RPMAs exhibit enhanced chemical and thermal stability, thereby extending the operating temperature range of water-soluble RPMA systems. Their relatively low molecular weight and superior injectivity allow them to penetrate deeper into tight or ultra-low permeability formations, where they help mitigate water blocking and improve gas-phase flow. Successful field deployments have been reported in the Natural Buttes gas field in Utah, USA [83], and the Dongpu condensate gas reservoir in Henan, China [84], where the use of fluorinated RPMAs led to effective water control and reduced water-lock damage.

#### 3.2.2. Adaptability of Relative Permeability Modification Agents in Gas Reservoirs

Relative permeability modification agent (RPMA) systems are characterized by low viscosity and high mobility, making them particularly suitable for applications in low-permeability and tight gas reservoirs as summarized in Table 5. However, their relatively weak mechanical strength and poor resistance to flow-back erosion limit their effectiveness under harsher reservoir conditions. Kalfayan et al. [85] suggested that RPMAs can be effective in gas wells with high water cut but are generally unsuitable for fractured reservoirs due to uneven fluid distribution and uncontrolled leakoff. According to Eoff et al. [6] and Botermans et al. [86], RPMAs perform best in stratified, homogeneous reservoirs where there is minimal crossflow between layers, no active water movement in hydrocarbon-producing zones, and distinct contrasts between high-permeability water-bearing layers and low-permeability gas-bearing layers. In such settings, significant disproportionate permeability reduction (DPR) effects can be achieved. Qi et al. [87] further emphasized that the effectiveness of RPMAs strongly depends on their adsorption behavior on the reservoir rock surface, particularly their interaction with surface charges. Therefore, optimizing the electrochemical compatibility between the RPMA and the rock matrix may enhance DPR selectivity.

Despite these advantages, several field studies have reported challenges such as delayed response time, low post-treatment gas production, and strong sensitivity to reservoir heterogeneity and operational parameters. To address these issues, RPMAs can be integrated with stimulation techniques, such as hydraulic fracturing or optimized using improved injection strategies, to enhance water-blocking selectivity and treatment efficacy. Furthermore, given their tendency to preferentially adsorb onto clay-rich, water-producing zones, the effective penetration depth of RPMAs may be limited [88]. To mitigate this, a preflush stage can be introduced to condition the formation before RPMA injection, thereby facilitating deeper placement and prolonging the agent’s effectiveness in water control.

### 3.3. Functional Fluids for Water Control

#### 3.3.1. Field Applications of Functional Fluids in Water Shutoff

Nanofluid-based water control agents have gained increasing attention due to their unique physicochemical properties, as summarized in Table 6. These agents include nanoemulsions, nano-active oils, and other functional colloidal systems. For example, Luo Mingliang et al. [89] developed a nanoemulsion composed of amino-polysiloxane and methyl ester sulfonate (MES) that showed good compatibility with tight reservoir pore-throat structures. Core flooding experiments indicated that the water-phase relative permeability was reduced by more than 60% after treatment. Sun Xiangyu et al. [90] formulated a nano-activated condensate fluid designed for high-temperature (140–150 °C), high-salinity (200,000 mg/L) edge and bottom-water condensate gas reservoirs. Core tests revealed that the post-treatment water production rate was reduced to one-ninth of the pretreatment level. Similarly, Yang Liping et al. [91] applied a nanoparticle-based active oil system in the Tarim Basin’s Tahe gas field, achieving a daily gas production increase of 70,000 m^3^ and a 75% reduction in water cut for a single well.

Importantly, organosilicon compounds dramatically alter capillary forces and imbibition rates, reducing the likelihood of water invasion into pore throats and effectively mitigating the “water-lock” effect. For example, Sun Houtai [92] developed a modified amino-silicone nanofluid with a median particle size of 30.2–84.2 nm that was matched to the pore-throat scale of low-permeability reservoirs. The system was thermally stable at 80 °C, reversed rock wettability from hydrophilic to hydrophobic, and reduced spontaneous imbibition by 27.2–31.3%, thereby supporting water control and gas stabilization. Another representative system is colloidal nanosilica fluids, which are composed of silica nanoparticles and activators. With particle sizes ranging from 3 to 100 nm and viscosities below 10 mPa·s, these systems can penetrate deep into water-bearing pore networks and transform in situ into high-viscosity or rigid phases, forming durable blockages [93].

For microemulsion-based systems, Yan Bo et al. [89] developed a high-temperature-stable, gas-wet emulsion for gas reservoirs at 106.7 °C. The formulation, which blended cetyltrimethylammonium chloride, stearyl trimethylammonium chloride, and modified imidazoline, achieved a water shutoff efficiency of 94.9% while maintaining gas flow. Field trials are also used to validate the potential of functional fluids. In the Alger gas field in Hungary, Lakatos et al. [94] employed a siloxane emulsion for water control, doubling gas output. In Kansas, USA, a latex concentrate composed of acrylamide derivatives, diesel/crude/condensate, emulsifiers, and water was used to treat water-producing zones. Post-treatment, water production was reduced to zero, and gas production reached 2831 m^3^/d. Additionally, in the Tunu gas field (Indonesia), a modified polyaluminum chloride functional fluid was applied in a water-producing formation. The intervention reduced water production by 90% while maintaining stable gas output.

#### 3.3.2. Adaptability of Functional Fluids in Gas Reservoirs

Functional fluids, characterized by their low viscosity and small particle size, are well-suited for use in low-permeability and tight reservoirs and have also shown promise in condensate gas reservoirs. However, enhancing their resistance to flow-back erosion remains essential to improving the longevity of water control performance. In the context of nanofluids, Foroozesh et al. [95] emphasized that one of their primary advantages lies in their durability under harsh reservoir conditions, including high temperature, high pressure, high shear, and high salinity, whereas conventional systems based on polymers and surfactants often degrade prematurely under similar conditions. Moreover, unlike microemulsion systems used in oil well treatments, which often undergo secondary in situ emulsification that may reduce selectivity [95], such phenomena are rarely observed in gas wells. As a result, microemulsions tend to retain better selectivity in gas production environments, making them particularly effective for water shutoff in water-producing gas wells.

## 4. Limitations and Technical Challenges of Water-Blocking Materials

Table 2 summarizes the key challenges and current technological maturity of polymer gel-based water shutoff in gas reservoirs from three perspectives: fundamental mechanism research, novel material development, and field-scale engineering applications. In terms of fundamental research, progress remains limited due to the complexity of multiphysics coupling, lack of in situ micro/nanoscale visualization, and difficulties in calibrating numerical models, leaving this area in the early stage of exploration. For material design, it is challenging to simultaneously achieve high thermal and salinity stability, deep reservoir penetration, and intelligent responsiveness. Most domestic efforts remain confined to laboratory-scale validations. Field applications, on the other hand, are hindered by issues such as “bypass” or “leapfrog” flow due to strong reservoir heterogeneity, lack of real-time monitoring and feedback control, and inconsistencies in injection processes. Although pilot-scale demonstrations have been conducted in some gas fields, concerns remain regarding scalability, operational stability, and economic feasibility. Taken together, the assessments in Table 2 and Table 7 indicate that although China has established a certain technical foundation in both fundamental and applied aspects of polymer gel-based water shutoff, large-scale industrial deployment will require further breakthroughs in mechanistic understanding, material industrialization, and digital construction control.

### 4.1. Fundamental Mechanistic Challenges

Research on the fundamental mechanisms of polymer-based water shutoff faces a series of complex and interrelated challenges. These difficulties arise not only from the intrinsic diversity of polymer molecular structures and physicochemical properties, but also from the multiscale heterogeneity of subsurface pore–fracture systems and the inherent limitations of current experimental and numerical modeling techniques. From a molecular perspective, polymer chains exhibit high flexibility and a wide range of conformations in solution [96]. The interaction between polymer segments and rock surfaces involves multiple forces, including electrostatic adsorption, van der Waals attraction, hydrogen bonding, hydrophobic interactions, and, in some cases, coordination or covalent bonding. These interactions are highly sensitive to temperature, salinity, pH, and ionic composition, thereby complicating extrapolation of laboratory adsorption data to real reservoir conditions. Additionally, polymer molecular weight, branching degree, and side-group functionality strongly influence the thickness, mechanical stability, and bridging capacity of the adsorbed layer. Determining the optimal molecular weight distribution remains a delicate balance among three competing requirements: pore size matching, interpore bridging, and deep formation penetration. A molecular weight that is too low weakens bridging capability, whereas excessively high molecular weight restricts injectivity.

The bridging and entrapment process itself is a cooperative, multichain phenomenon. Polymer segments may simultaneously adsorb on opposing pore walls, forming “single-chain bridges” or “multichain network bridges.” The initiation and strength of these bridges depend on pore geometry, local flow velocity, shear stress, polymer concentration, and injection rate [97]. Capturing the onset location, bridge volume, and propagation dynamics of such networks at the micro- and nanoscale remains extremely challenging. Although microfluidic visualization combined with fluorescence microscopy, nano-CT, or Raman imaging can partially reveal localized behaviors, they fail to capture the full multiscale evolution from nanometers to millimeters or centimeters, preventing the establishment of a unified mechanistic framework.

At the crosslinking and gelation stage, chemical reaction kinetics further complicate the picture. The stoichiometric ratio, activation energy, and reaction rate between crosslinkers and polymer functional groups are strongly affected by temperature, pH, salinity, and multivalent ion concentration. Conventional batch experiments or static rheological tests cannot accurately reproduce the coupled flow, non-isothermal, and non-isobaric conditions of real reservoirs. Premature gelation near the wellbore can cause plugging, whereas delayed gelation in deeper zones may lead to insufficient sealing [90]. Achieving a proper balance between reaction kinetics and transport distance remains a central challenge in both laboratory optimization and field-scale implementation. Moreover, the mechanical coupling between the carrier fluid and the polymer network introduces additional complexity. Polymer gels are viscoelastic materials whose storage and loss moduli vary dramatically under different shear frequencies and stress amplitudes. A gel must be elastic enough to resist pressure differentials and shear forces, yet viscous enough to dissipate energy and restrict water mobility. However, standard rheometers cannot replicate the wide range of shear rates and chemical conditions encountered in situ, making it challenging to extrapolate laboratory-derived rheological curves to real-field performance.

Even after gel formation, the relative permeability modification mechanism remains poorly understood. How do the flow pathways of water and gas (or oil) evolve dynamically once gel occupies part of the pore network? Which pore size range contributes most effectively to selective water blocking without impeding gas flow? How can partial gas connectivity be preserved? Current micromodel and coreflood experiments, combined with macroscopic productivity evaluation, cannot yet provide generalizable conclusions. Progress will require coupling multiphase flow simulations, pore network models, and direct microscopic observations for comprehensive validation. From a numerical modeling perspective, most current polymer–porous medium coupling models rely on simplified extensions of Darcy’s law or empirical filtration equations, often neglecting adsorption gradients, nonlinear bridge geometries, crosslinking kinetics, and higher-order couplings between reaction and flow fields. Consequently, discrepancies persist between model predictions and experimental or field observations. Realistic simulations demand both high-performance computing frameworks and extensive high-precision experimental datasets for constitutive model calibration—resources that are often constrained by cost and time [98].

Furthermore, the geological heterogeneity of subsurface formations, including fracture orientations, pore connectivity, and permeability contrasts, introduces strong randomness and uncertainty into polymer propagation. Reproducing such stochastic conditions in laboratory settings or incorporating geological statistical methods into models for accurate representation remains a major bottleneck for mechanistic studies.

Finally, although numerous emerging technologies have been applied for microscale characterization and macroscale monitoring, these approaches often operate in isolation. The lack of a unified data processing and multimethod integration framework has resulted in fragmented insights that are difficult to consolidate into a coherent understanding [99,100]. This fragmentation limits the translation of mechanistic knowledge into practical guidance for material innovation and field-scale design.

In summary, research on the fundamental mechanisms of polymer-based water shutoff urgently requires breakthroughs in multiscale experimentation, multiphysics coupling, and integrated modeling. Establishing a cohesive, data-driven framework that bridges molecular-level interactions with field-scale flow dynamics is essential to achieving accurate prediction, guiding the development of next-generation high-performance polymers, and enabling intelligent water shutoff strategies in complex gas reservoirs.

### 4.2. Material Design Limitations

#### 4.2.1. High-Temperature/High-Salinity (HTHS) Resistant Monomers

In the field of polymer material design for water shutoff, one of the foremost challenges lies in ensuring thermal and salinity stability and long-term aging resistance of materials under harsh reservoir conditions. In unconventional gas reservoirs, formation temperatures often reach 120–160 °C or higher, and salinity levels frequently exceed 106 mg/L [101]. Conventional gel systems based on polyacrylamide (PAM) or polyacrylic acid (PAA) tend to suffer from chain scission, thermally induced degradation, ion exchange-driven structural collapse, and premature dehydration, all of which severely compromise their adsorption capacity, bridging behavior, and crosslinked network integrity, ultimately reducing mechanical strength. To overcome these limitations, researchers have explored copolymer modification strategies, introducing multifunctional monomers such as thermo-responsive units (e.g., N-isopropylacrylamide), salt-tolerant monomers (e.g., sodium acrylate), and silane coupling agents to optimize the polymer backbone. However, balancing thermal stability with deep formation penetration remains a critical design dilemma. Although increasing backbone rigidity or crosslinking density enhances thermodynamic stability, it often comes at the cost of reduced injectivity and limited migration depth, highlighting the delicate trade-off inherent in designing high-performance polymer systems for extreme subsurface environments.

#### 4.2.2. Molecular Weight Control for Optimized Injectivity and Performance

Precise control over molecular weight plays a critical role in optimizing polymer performance. Insufficient molecular weight may result in poor bridging capability, thin adsorption layers, and weak network strength. Conversely, an excessively high molecular weight hinders migration through micron-scale pore throats, leading to premature accumulation and plugging near the wellbore. To balance injectivity and bridging effectiveness, researchers have explored strategies such as broad molecular weight distribution, branched architectures, and even star-shaped or hyperbranched polymers [81]. However, the synthesis of such complex macromolecular structures is often technically demanding and cost-intensive. Additionally, increased branching introduces higher sensitivity to impurities and batch-to-batch variation, posing challenges to manufacturing consistency. To further improve shear resistance and mechanical integrity, nanocomposite reinforcement has emerged as a promising avenue. By incorporating nanoparticles such as nanosilica, nanoclay, carbon nanotubes, or hydroxyapatite into the gel matrix, a hybrid “skeleton–colloid: structure can be constructed at the molecular level, significantly enhancing the gel’s elastic modulus and long-term durability [102,103]. However, ensuring uniform dispersion of nanoparticles within the three-dimensional polymer network—without agglomeration—while simultaneously achieving strong interfacial interactions with polymer chains imposes stringent demands on surface functionalization and dispersion techniques.

#### 4.2.3. Smart Responsive Systems

In addition, the design of smart stimuli-responsive polymer systems presents significant challenges. Thermo-sensitive, pH-sensitive, and salinity-sensitive polymers are required to delay gelation until after deep reservoir penetration. However, the trigger thresholds—whether temperature or pH—must be precisely defined, often within just a few degrees Celsius or minimal pH units [104,105]. Any deviation may result in premature gelation near the wellbore or insufficient activation in the far-field, leading to ineffective treatment. Moreover, developing multi-stimulus-responsive polymers (e.g., systems simultaneously activated by temperature, pH, and salinity) requires incorporating multiple responsive functional groups within the same polymer backbone or within a mixed formulation. This complexity increases the difficulty of molecular design and synthesis, while also enhancing the system’s sensitivity to reservoir heterogeneity and the complex composition of injection fluids, thereby introducing greater uncertainty in both laboratory scale-up and field deployment.

From a practical perspective, the design of new functional materials must also account for engineering feasibility and economic viability. Regardless of whether nanocomposite reinforcement, microcapsule-based controlled release, or multiresponsive functionalization is employed, the material system must be compatible with conventional drilling fluids, spacer fluids, and injection equipment and maintain stability during mixing and transport under field conditions. Although many high-performance systems show promise under laboratory conditions, scale-up challenges often arise due to expensive raw materials, complex synthesis procedures, and a large number of required additives. This is especially problematic in gas wells with large borehole volumes or extended horizontal sections, where injection volumes may reach several tons per well [105]. Under such conditions, material cost and operational risk become major bottlenecks limiting widespread application and commercialization.

#### 4.2.4. Green and Environmentally Friendly Materials

Environmental and safety considerations have become indispensable aspects of modern material design. Conventional crosslinkers—particularly those based on chromium ions—pose potential environmental toxicity risks [106]. To achieve thermal and salinity resistance, metal ligand-based crosslinking systems often require higher concentrations of heavy metal ions, which may accumulate in the formation and adversely affect groundwater quality or subsequent hydraulic fracturing operations. Therefore, the development of green, low-toxicity, and biodegradable crosslinking systems that maintain long-term stability under reservoir conditions remains a critical challenge for researchers.

Lithological variability also significantly influences material performance. Different reservoir rock types, such as sandstone, carbonate, and shale, exhibit distinct mineral compositions and surface chemistries, leading to substantial differences in polymer adsorption, bridging behavior, and overall shutoff efficiency [107,108]. As a result, universal material formulations often fail to perform consistently across diverse geological settings. This necessitates extensive formation-specific coreflood experiments, as well as binary and ternary system evaluations, further increasing the complexity and cost of material screening and design workflows. Finally, in the era of digitalization and intelligent engineering, material development faces the challenge of integrating with numerical simulation, machine learning, and digital twin technologies. A forward-looking approach aims to simulate material behavior through multiphysics platforms during the design stage, followed by experimental validation and field-scale calibration, thus establishing an efficient “simulate–experiment–deploy” workflow [109,110]. However, at the material level, the lack of standardized high-throughput characterization datasets and uniform performance evaluation metrics hampers the implementation of data-driven design paradigms and the broader industrial adoption of intelligent materials.

### 4.3. Engineering and Field-Scale Constraints

Although polymer gel-based water shutoff technologies have shown promising results in laboratory studies and pilot-scale trials, their large-scale application in heterogeneous and unconventional gas reservoirs still faces multiple complex challenges. First, reservoir heterogeneity significantly influences the migration and placement of polymer precursor fluids [111]. In fracture–matrix composite systems, high-permeability channels are preferentially accessed, leading to near-wellbore gelation and insufficient penetration into low-permeability or stagnant zones. This results in inefficient shutoff and risks such as wellbore plugging or abnormal injection pressure, commonly referred to as “by-pass” or “skipped zones.”

Second, downhole conditions vary drastically compared to the laboratory. Reservoir temperatures may surge from 50 °C to over 120 °C, and salinity can exceed 100,000 mg/L. Under such conditions, even small deviations in trigger thresholds (e.g., temperature or pH) can result in premature gelation near the well or delayed crosslinking in the far-field, both of which compromise the shutoff integrity. Currently, the lack of a real-time feedback mechanism between injection parameters and formation response hinders dynamic control [112,113]. Although resistivity imaging and acoustic logging have been introduced for on-site monitoring, these methods generally reflect only the approximate gel penetration depth and cannot precisely quantify pore occupancy or gel continuity, making real-time optimization of injection rate, concentration, and crosslinker ratio challenging.

Third, on-site material preparation and mixing pose logistical challenges. Typical gel precursors require precise blending of polymers, crosslinkers, retardants, and other additives. However, field mixing equipment often struggles to replicate laboratory formulations accurately. Moreover, during bulk storage and long-distance pumping, shear degradation or premature crosslinking may occur, adversely affecting injectivity and penetration performance.

In addition, increasing environmental and safety regulations have imposed stricter limits on the use of heavy metal-based crosslinkers such as chromium or aluminum. There is an urgent need to develop green, low-toxicity, and biodegradable alternatives with comparable durability and controlled breakdown capability. However, many of these new eco-friendly systems still lack sufficient field validation under high-temperature, high-salinity, and fractured reservoir conditions.

Lithological diversity further complicates performance prediction. Variations in mineral composition, pore structure, and surface chemistry across sandstone, carbonate, and shale formations cause significant differences in polymer adsorption, bridging, and gel formation behavior. As a result, a “one-size-fits-all” formulation is often ineffective. Instead, extensive formation-specific coreflooding and multicomponent compatibility testing are needed, which increase project timelines and operational costs. Moreover, a delicate balance must be maintained between performance enhancement and economic feasibility [114]. Although advanced polymers, such as stimuli-responsive systems, nanocomposite-reinforced gels, and microcapsule-based delivery technologies, can improve shutoff efficiency, they also increase material costs and field complexity. In horizontal wells with long lateral sections, injection volumes may reach several to tens of tons, making cost control critical. Additionally, multizone segmented injection requires precise control of gel formulation, viscosity, and molecular weight at each stage, and avoiding crossflow or intermixing between layers demands sophisticated downhole isolation and co-injection technologies.

Finally, ensuring long-term stability and reversible reopening capability of the gel is essential. Field monitoring has demonstrated that some wells experience strength degradation within one year post-treatment, leading to partial water breakthrough. Furthermore, prefracturing or acidizing operations require effective gel breakdown, and improper selection of breakers, pH triggers, or timing may adversely affect stimulation results.

In summary, achieving efficient, durable, controllable, and cost-effective polymer gel shutoff in gas reservoirs requires coordinated advancements in several domains: reservoir heterogeneity analysis, injection monitoring integration, formulation–operation consistency, green crosslinking systems, lithology compatibility, cost-performance optimization, and dual-functionality design for long-term stability and reversibility. Addressing these interconnected challenges is essential for the scalable deployment of polymer gel technologies in deep, heterogeneous, and unconventional gas reservoirs.

### 4.4. Cross-Category Comparison and Material Selection Framework

A recurring reason for inconsistent field outcomes is the mismatch between material mechanism and reservoir-specific constraints. Therefore, we propose a practical selection workflow based on three filters: (i) flow-path scale (fracture/channel vs. tight matrix), (ii) harsh environment tolerance (temperature/salinity/CO_2_), and (iii) selectivity requirements (preserve gas while restricting water).

For fracture-dominated channels requiring high differential pressure resistance, in situ crosslinked gels, gel–foam hybrids, and particle/microgel blends are preferred. For tight matrices where injectivity dominates, nanoemulsions, microemulsions, and RPMAs with strong adsorption but low viscosity are more suitable, often preceded by a preflush.

To make this decision process operational, future reports should consistently provide a minimum metric set that includes temperature/salinity, permeability and fracture characterization, injection pressure window, water-phase permeability reduction, gas-phase impairment, and longevity under dynamic flow-back.

## 5. Future Directions

### 5.1. Emerging Water Shutoff Materials

The future development of water shutoff materials will focus on multifunctional integration, environmental compatibility, and intelligent responsiveness [115,116]. Material design is expected to move beyond traditional single-polymer systems toward synergistic multicomponent composites, such as polymer–nanoparticle hybrids, surfactant-enhanced gels, and microcapsule-based systems, aiming to balance sealing strength, injectivity, and long-term durability. In response to increasingly complex reservoir conditions, such as high temperature, high salinity, high pressure, and multiscale fracture–pore networks, materials must exhibit enhanced thermal stability, shear resistance, and interfacial adaptability, enabling broad compatibility with various gas reservoir types.

#### 5.1.1. Thermal- and Salt-Resistant Materials

In deep gas reservoirs, formation conditions are typically extreme, with temperatures exceeding 100 °C and total dissolved solids (TDSs) greater than 10^5^ mg/L. The high concentrations of divalent ions such as Ca^2+^ and Mg^2+^ can readily induce polymer precipitation, ion exchange, and thermal degradation. Conventional hydrolyzed polyacrylamide (HPAM) and its copolymers suffer rapid viscosity loss and crosslinking failure under such conditions, resulting in unstable gel structures and poor sealing performance. Consequently, the development of next-generation polymeric materials with exceptional thermal and salinity tolerance has become a critical research focus in gas reservoir water shutoff technology [117,118].

Recent studies have demonstrated that introducing heat-resistant functional groups, sulfonic or cationic monomers, and supramolecular or bio-based structural motifs can significantly enhance polymer stability and gel strength under harsh conditions, thereby improving deep reservoir sealing performance. Drawing inspiration from enhanced oil recovery (EOR) and fracturing fluid chemistry, many researchers have focused on designing copolymers containing sulfonic or zwitterionic monomers combined with rigid structural units to suppress chain contraction and maintain molecular extension at high temperatures. For example, Liu et al. [46] synthesized a binary copolymer (PDMT-1) by incorporating zwitterionic and bulky cyclic maleic anhydride units into the PAM backbone. The material maintained high viscosity and structural integrity under conditions of 110 °C and 75,000 mg/L salinity, enabling large-scale field applications and significantly improving production rates.

In parallel, non-covalent crosslinking strategies have gained attention for improving thermal stability and viscoelastic performance. Peng et al. [47] developed a β-cyclodextrin-based host–guest supramolecular hydrogel employing rigid dual-tail hydrophobic monomers. The reversible hydrophobic association within the β-cyclodextrin cavity and dynamic dual-tail aggregation formed a stable non-covalent network exhibiting excellent thermal stability up to 120 °C, with superior viscosity retention and enhanced shear resistance compared with HPAM [50].

Furthermore, the design of functionalized copolymers and nanocomposite networks is becoming a dominant trend. Guo et al. [48] reported a selective gel system for a 110 °C, 2.24 × 10^5^ mg/L high-salinity gas reservoir based on AM–AMPS copolymers crosslinked with p-benzenediol and hexamethylenetetramine and stabilized using thiourea antioxidants. The resulting gel achieved over 98% reduction in water-phase permeability within 0.5 PV injection and exhibited effective conformance control between matrix and fractures. Li et al. [49,50] further advanced this concept by developing an intelligent recrosslinkable hydrogel using dialdehyde cellulose nanofibers (DA-CNF), polyvinyl alcohol (PVA), and tannic acid (TA). At 130 °C and 1.0 × 10^5^ mg/L salinity, the gel maintained high stability, forming a secondary reinforcement network via TA-metal coordination and dynamic hydrogen bonding [119]. The thermosensitive PVA component exhibited “low-temperature contraction for injectivity” and “high-temperature expansion for plugging,” providing precisely controllable sealing behavior for deep reservoirs.

Beyond organic systems, inorganic and hybrid water shutoff agents also show promising thermal and salinity resistance. Silicate-, phosphate-, and aluminosilicate-based gels can withstand extreme ionic strengths and high temperatures while forming rigid, self-healing mineral frameworks. Colloidal silica, nano-SiO_2_, and clay-based nanogels possess low viscosity, high mobility, and excellent resistance to electrolyte interference, making them ideal candidates for deep penetration and in situ solidification. In addition, nanohybrid composites combining polymeric matrices with inorganic fillers (e.g., graphene oxide, montmorillonite, or hydroxyapatite) provide structural reinforcement through hydrogen bonding and electrostatic interactions, effectively increasing mechanical robustness and longevity under harsh thermal and saline environments.

Looking forward, the future of high-temperature- and high-salinity-resistant materials lies in the integration of molecular-level design, supramolecular engineering, and nanostructure optimization. Strategies such as functional monomer synergy, dynamic crosslinking networks, bio-inspired fiber reinforcement, and temperature-controlled swelling systems will continue to drive the evolution of polymer-based plugging agents from conventional HPAM gels toward high-strength, multifunctional, and intelligent adaptive systems. In addition, inorganic and nanocomposite materials will complement organic polymers, jointly expanding the operational envelope for water shutoff technologies in deep, heterogeneous, and ultra-high-salinity gas reservoirs.

#### 5.1.2. Easily Injectable Water Shutoff Agents

In chemical water shutoff operations for gas reservoirs, the injectability of polymer-based plugging agents is severely constrained by the dual limitations of low reservoir permeability and microscale pore-throat structures. Most conventional polymers possess high molecular weights, with hydrodynamic radii comparable to pore-throat diameters, making them prone to bridging, adsorption, and accumulation near the wellbore [120]. This leads to the formation of filter cakes, sharp increases in injection pressure, and a significantly reduced effective radius, ultimately impairing deep migration and large-scale blocking efficiency. Additionally, the strong surface adsorption of polymers, though enhancing blocking strength, further reduces matrix permeability and restricts long-distance propagation. In high-temperature and high-salinity reservoirs, polymer chains tend to undergo salting-out and thermal degradation, reducing the injection-to-retention ratio and creating a narrow operational window characterized by “injectable but non-migratable” behavior [121].

To overcome these challenges, researchers are working to optimize polymer molecular weight, chain architecture, and functional modification to ensure deep penetration while maintaining blocking performance. Supramolecular self-assembling polymers have emerged as a promising solution, offering injectability without relying on high molecular weights. These polymers form dynamic, reversible networks through non-covalent interactions (e.g., host–guest recognition, hydrogen bonding, hydrophobic associations). Such dynamic structures can depolymerize under high shear (e.g., during pore throat traversal) to reduce flow resistance and then spontaneously reassemble into three-dimensional gel networks in situ, thereby combining injectability with effective sealing. For example, Zhang et al. designed a supramolecular network based on guanine–cytosine (G≡C) triple hydrogen bonds grafted onto HPAM chains, which reduced the injection pressure by two-thirds compared with conventional HPAM in 15 mD core samples [122]. In another study, a dual-network polymer system comprising β-cyclodextrin-modified HPAM and adamantane-functionalized welan gum demonstrated superior injectability and blocking efficiency under high temperature and salinity conditions, achieving 12–17% recovery enhancement in low-permeability cores. Moving forward, injectable material development will extend beyond traditional polymers and focus on several strategic directions:

(1) Low-viscosity and transformable non-polymeric systems: These systems include functional nanofluids, emulsifiable oil-based agents, and nanoemulsions that exhibit small particle size and low viscosity for easy injection. These systems can undergo in situ transformation into effective plugging materials under formation conditions. These are suitable for tight, fractured, or ultra-low permeability gas reservoirs.
(2) Trigger-responsive microstructured systems: These systems include pH-sensitive, thermo-responsive, and salt-responsive microgels or sol–gel precursors that remain injectable during placement and undergo structural transformation upon reaching target zones, balancing injectability with selectivity.
(3) Programmed and synergistic injection strategies: These strategies employ intelligent design of injection slugs, viscosity gradients, and real-time monitoring (e.g., pressure drop, resistivity) to guide agent placement toward dominant water-bearing pathways while dynamically adjusting injection parameters, thus forming a closed-loop “injection–response–control–sealing” mechanism.
(4) Eco-friendly and degradable systems: In response to environmental and regulatory pressures, the development of green, low-toxicity, and biodegradable materials, such as modified natural polymers, small-molecule self-assembling gels, and biocompatible reversible crosslinking networks, offers potential for integration into long-term reservoir management schemes.

In summary, the evolution of injectable materials is shifting from viscosity control to a broader paradigm encompassing structural adaptability, stimulus responsiveness, environmental compatibility, and intelligent deployment. Tailoring material design to match reservoir heterogeneity, pore-throat distributions, and dynamic water invasion mechanisms will be critical to achieving precise and large-scale chemical water shutoff in deep and complex gas reservoirs.

### 5.2. Multi-Level Water Shutoff Materials

In complex and heterogeneous gas reservoirs, a single chemical water shutoff system often fails to simultaneously meet the control requirements for both fractures and matrix flow channels [123,124]. On one hand, high-permeability fractures and macropore networks demand the rapid formation of robust, low-mobility gels to establish a stable plugging barrier. On the other hand, the dense matrix with micron-scale pore throats requires highly injectable chemical agents capable of deep penetration to achieve effective water control. To address this contradiction, this paper proposes a multisystem zonal cooperative water shutoff strategy. Specifically, high-strength in situ crosslinked gels or preformed microcapsules are first injected into fracture-dominated or high-permeability zones to seal dominant water pathways. Subsequently, nano-/microemulsion-based relative permeability modifiers or supramolecular adaptive polymers with excellent injectability are introduced into matrix zones to regulate water flow within the fine pore network. By carefully designing the sequence of segmented isolation and staged injection, interference between agents can be minimized, achieving a balance between plugging strength and deep penetration.

#### 5.2.1. Macroscale Plugging Combined with Microscale Flow Regulation

In heterogeneous gas reservoirs, permeability and pore-throat structures vary significantly, with high-permeability fractures coexisting alongside low-permeability matrix zones. Conventional water shutoff agents often struggle to balance injectivity and plugging strength under such conditions. In fracture zones, strong gels are needed to block major water channels; however, they tend to hinder deep penetration into the matrix. In contrast, low-viscosity microscopic agents are required to enter the tight matrix, but they typically lack sufficient strength after plugging [125]. High-temperature and high-salinity conditions further exacerbate polymer degradation and salting-out failures, whereas limited real-time monitoring makes it difficult to evaluate plugging depth and uniformity during field operations, thus posing major challenges to precise chemical water shutoff.

A “macroscopic plugging and microscopic permeability regulation” strategy offers promising dual-scale synergy for controlling water invasion. First, high-strength in situ crosslinked gels or preformed microgel particles are injected into large pores and fractures to rapidly form stable barriers at the macroscale, effectively sealing dominant water flow paths and preventing agent loss. Next, low-viscosity microemulsions or supramolecular adaptive polymers are introduced into the tight matrix; these materials, with particle sizes less than 100 nm and dynamic reversible crosslinking capabilities, can deeply penetrate pore throats as small as 0.1 mD, achieving fine-scale plugging of residual pore space while minimally affecting gas-phase flow [126,127,128]. This dual approach addresses both sealing efficiency and deep injectivity. With segmented zonal isolation and pressure gradient-controlled injection, the method avoids the limitations of single systems that either “fail to propagate” or “block gas pathways”.

Extensive research has demonstrated that gel particles exhibit strong water-phase swelling while minimally responding to oil/gas, enabling preferential water-phase blocking after field injection. For instance, Kawelah et al. (2015) [125] proposed a high-temperature resistant polyacrylamide–foam hybrid for fractured gas reservoirs at 150 °C. Alternating injections of gel precursors and foam enabled foam-assisted delivery of the gel into large channels, followed by in situ curing to form a dense gel–foam barrier. Results showed that after injecting 0.5 PV, water-phase permeability decreased by more than 95%, whereas gas-phase permeability was reduced by less than 20%, confirming the system’s potential for fracture–matrix cooperative water control in high-temperature, high-salinity, and heterogeneous gas reservoirs.

In water shutoff applications for tight matrix zones, researchers are exploring novel chemical agents with both deep injectivity and effective blocking performance. Hu Anbang et al. developed a polylactic acid (PLA)-based high-density shutoff material soluble in natural gas. Upon contact with bottom water, it rapidly solidifies into a strong plugging layer, cutting off water inflow. Then, in a methane environment, it undergoes self-degradation, restoring fracture conductivity and achieving dual “water shutoff–gas conduction” functionality. Experiments showed 100% curing within 4 h at 90 °C, with a gas-phase degradation rate of 97.6% and negligible impact on gas permeability during post-treatment gas drive, demonstrating excellent injectivity and selective water control [126].

Combining such low-permeability water control agents with high-strength in situ gels or microgel particles previously applied in fractures enables “dual-zone cooperative water shutoff”. First, high-strength gels rapidly seal high-permeability conduits; then, emulsified or responsive agents penetrate microporous networks to build a full-scale sealing system. This strategy has the potential to overcome the long-standing bottleneck of poor compatibility between injectivity and plugging performance in chemically treated heterogeneous gas reservoirs.

#### 5.2.2. Suggested Injection Schedule and Multistage Isolation Workflow

In the multisystem zonal cooperative water shutoff strategy, to ensure that each chemical plugging agent performs optimally within its designated seepage channel, the injection and zonal isolation process should follow a five-step sequence: pretreatment → plugging of high-permeability zones → supplementary treatment of medium–low-permeability zones → evaluation and adjustment → final zonal isolation [129]. First, a low-viscosity pretreatment fluid, such as a surfactant-containing flushing solution or a weak acid, is injected to modify the reservoir wettability, remove near-wellbore blockages, and reduce injection resistance, thereby preparing the formation for the deep migration of subsequent polymeric or nanomaterial agents. Next, high-strength in situ crosslinked gels or preformed microcapsules are injected into fracture-dominated or ultra-high-permeability zones using sliding sleeve packers for precise zonal placement. These agents rapidly form robust barriers within wide fractures and large pores, effectively cutting off the dominant water pathways. After injection, a controlled pressure that is maintained below the designed fracture pressure should be applied to prevent gel backflow and preserve the structural integrity of the network. In the third stage, the packer is released, and injection is switched to the medium-low permeability intervals, where low-molecular-weight relative permeability modification agents or supramolecular self-assembling polymers are introduced. These low-viscosity, nanoscale agents can penetrate dense matrix pores down to 0.1 mD and achieve controlled deep plugging through adsorption or wettability alteration, while maintaining gas-phase permeability [130]. The fourth step involves performance evaluation and adaptive adjustment, combining production pressure differential monitoring with tracer-based diagnostics. Using surface sampling, tracer response analysis, and zonal pressure monitoring along the casing, the sealing efficiency and retention ratio of each interval are evaluated in real time. If backflow in high-permeability zones or insufficient sealing in low-permeability zones is detected, targeted reinjection or agent replacement can be promptly implemented. Finally, after all injection stages are completed, reverse operations using packers or sliding sleeves restore the full gas production pathway, leaving behind only a “chemical barrier” at the interfaces between high-permeability layers and the dense matrix. This enables phase-diverted flow, with water and gas moving through separate pathways, while maintaining unobstructed wellbore production [131].

This procedure ensures strong water blocking in high-permeability zones and effective deep penetration in tight formations, while allowing flexible control through adjustable pressure differentials and zonal isolation technology. The approach avoids the dual pitfalls of single-agent systems—either “injectable but shallow” or “over-plugged with production loss”—and provides a comprehensive, controllable framework for efficient chemical water shutoff in heterogeneous gas reservoirs.

### 5.3. Summary

The ultra-high temperature and salinity resistance of functional monomer-modified polymers and host–guest supramolecular gel systems enables them to maintain high viscosity and structural integrity under harsh reservoir conditions, such as temperatures up to 120–130 °C and salinity exceeding 2 × 10^5^ mg/L. For example, a low-molecular-weight freeze gel based on AM–AMPS copolymer and resorcinol/hexamethylenetetramine retains over 98% reduction in water-phase permeability at 110 °C and 2.24 × 10^5^ mg/L salinity. Similarly, a dual-network supramolecular polymer system (XG-AD and HPAM-CD) exhibits a viscosity retention rate of up to 80% under temperatures ranging from 25–130 °C in high-salinity environments, while also achieving self-healing through dynamic supramolecular assembly [132]. Such materials are ideally suited for sealing high-permeability water channels in high-temperature, high-salinity gas reservoirs.

In contrast, nanofluid/microemulsion-based relative permeability modifiers and supramolecular adaptive polymers offer excellent injectivity into tight matrix zones (0.1–10 mD) while minimally affecting gas-phase flow, making them optimal for fine-scale permeability regulation in compact formations. In addition, intelligent response and controlled delivery features—developed in other domains—present promising avenues for future material development. For example, pH-, temperature-, or CO_2_-responsive polymers can achieve dual functionalities: low-viscosity/high-permeability injection during placement and high-strength sealing post-deployment. These smart materials are especially well-suited for multistage or zonal plugging in ultra-heterogeneous gas reservoirs.

In summary, emerging polymeric materials featuring extreme environmental tolerance, deep injectability, high sealing strength, and intelligent responsiveness enable full-scale zonal water control from high-temperature fractured zones to tight matrix segments. These innovations offer customizable and diverse solutions for future chemical water shutoff operations in complex gas reservoirs. The zonal cooperative water shutoff strategy achieves dual control over water influx by strategically applying high-strength gels and easily injectable regulators in respective seepage pathways. This approach offers three main advantages:

1. Improved plugging efficiency: In high-permeability fractured zones, in situ crosslinked gels quickly form robust barriers, preventing early agent retention in matrix zones and enabling effective use of deep-penetrating materials.
2. Reduced chemical consumption: Zonal injection significantly reduces the required dosage of expensive functionalized polymers, enhancing economic viability.
3. Optimized flow profile: Following zonal plugging, production pressure differentials are more likely to form across tight matrix segments, promoting uniform displacement and sustained production enhancement.

For effective implementation, the following key practices are recommended:

♦ Accurate zonal delineation: Utilize logging data, tracer diagnostics, and formation pressure responses to define fracture–matrix interfaces and configure packer placement accordingly.
♦ Sequential injection and pressure gradient control: Begin with low-pressure injection of microemulsions or supramolecular polymers in matrix zones, followed by slightly higher-pressure injection of high-strength gels into fractured zones. This avoids cross-contamination and ensures spatial selectivity.
♦ Online monitoring and dynamic adjustment: Use tubing pressure differentials, surface production data, and tracer concentrations for real-time feedback. Adjust injection ratios and rates as needed to optimize placement.
♦ Compatibility verification: Prior to field deployment, conduct coreflood experiments to assess chemical compatibility and interactions among different systems. This prevents phase separation, undesirable reactions, and injection blockages.

Through coordinated optimization of these key elements, the zonal cooperative water shutoff strategy provides enhanced precision and engineering controllability in managing water production in highly heterogeneous gas reservoirs.

### 5.4. Cost, Sustainability, and Regulatory Considerations

Beyond performance, cost and environmental compatibility increasingly determine field adoption. Compared with conventional polyacrylamide-based gels, nanocomposite and supramolecular systems often introduce specialty monomers, nanoparticles, or fluorinated surfactants that increase material cost and may trigger stricter EHS scrutiny. Future work should report cost drivers (dosage, unit price, logistics), toxicity/biodegradability screening, and produced water treatability, enabling a realistic techno-economic and regulatory assessment alongside plugging performance.

## 6. Conclusions

**Figure 1:**
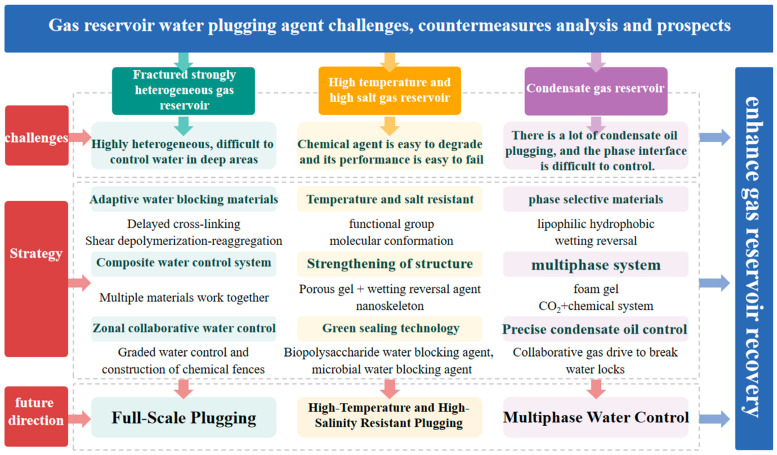
Gas reservoir water plugging challenges, countermeasures analysis and prospects.

In gas reservoirs with strong fracture-induced heterogeneity, severe permeability contrasts and uncontrollable deep water channels demand the development of delayed-crosslinking and precisely triggered adaptive shutoff materials, as well as multiagent cooperative systems to construct a full-scale plugging strategy. In high-temperature and high-salinity reservoirs, the rapid degradation and performance failure of conventional shutoff agents necessitate a shift toward molecularly modified polymers and advanced network architectures with enhanced thermal and saline tolerance. Future research should also explore biomimetic foams and nano-reinforced frameworks to achieve engineering-ready sealing systems under extreme conditions. For condensate gas reservoirs, characterized by condensate accumulation and complex phase interfaces, oil-affinitive and hydrophobic materials with interfacial activity are required to enable selective oil-phase shutoff. These should be used in conjunction with foam–gel hybrid systems or CO_2_-assisted chemical shutoff technologies and integrated with intelligent triggering mechanisms and numerical simulation platforms to precisely control the condensate behavior (Figure 1). Altogether, these technical directions are converging into three major water shutoff technology systems:

♦ Full-scale conformance control systems targeting multiscale heterogeneity;
♦ Thermal- and salinity-resistant shutoff systems for harsh reservoir environments;
♦ Multiphase selective shutoff technologies designed for complex gas–liquid systems.

These systems collectively aim to provide comprehensive technical support for significantly enhancing gas reservoir recovery efficiency.

## Figures and Tables

**Table 1:** The advantages and disadvantages of water shutoff agents, their gas reservoir adaptability, and supporting technologies. Plugging strength refers to the maximum sustained differential pressure reported in representative lab/core tests or field evaluations; values are compiled from the cited references in Section 2 and Section 3 and should be interpreted as indicative ranges rather than universal limits.

Type of Water Shutoff Agent	Mechanism	Advantages	Application Limitations	Gas Reservoir Adaptability	Plugging Strength	Supporting Technologies
Inorganic Salts and Resins	In situ precipitation/curing	High temperature resistance (up to 150 °C); strong salt resistance; high plugging strength (maximum 24 MPa); suitable for high-permeability and large-scale water shutoff [25,26,27]	Short migration distance; difficult to reach deep formations; poor adaptability to fracture systems	High-permeability gas reservoirs	Up to ~24 MPa (reported) [25,26,27]	Precise water-finding technology; targeted delivery technology of water shutoff agents
Particles	1/3 particle size matching and physical bridging	Low cost; salt resistance (200–300 g/L); temperature resistance (25–200 °C)	Weak migration ability; prone to cause plugging in near-wellbore areas; not suitable for deep and complex fractures	Near-wellbore areas; gas reservoirs with a temperature range of 25–200 °C and salt content of 200–300 g/L	Up to 10 MPa	Pore-throat matching technology
Polymer Adsorption Bridging Agents	Polymer molecules adsorb onto pore walls and form dense filter cakes through bridging	Good selectivity; strong migration ability	Limited strength; requires chemical crosslinking agents to cope with high pressure differences	Condensate gas reservoirs; low-permeability reservoirs; deep-water plugging; applicable at 150 °C with a salt content of 250–330 g/L	<5 MPa	Pretreatment fluid and fracturing bridging
Polymer Gels	Chemical crosslinking and physical interception/adsorption	High plugging strength; high temperature resistance; salt resistance; can be designed with gas channels or prefabricated microgels; suitable for fractures, high-porosity and high-permeability reservoirs, and deep-water plugging	Requires crosslinking agents; high cost; high injection pressure; prone to cause plugging in near-wellbore areas	Applicable at 30–150 °C and with salt content of 5–30 g/L	10–15 MPa	Pretreatment fluid and delayed crosslinking
Wettability Modifiers	Change the wettability of rock surfaces (from hydrophilic to hydrophobic)	Good migration; good selectivity	Dependent on rock mineral composition and permeability; significantly affected by pH and minerals	Low-permeability reservoirs; reservoirs with obvious gas–water interfaces	——	——
Foams	Jamin effect	Good plugging effect on large fractures/channels; selective water shutoff	Sensitive to high temperature and high salt; poor stability in high-temperature gas reservoirs (temporary plugging)	Poor stability in high temperature gas reservoirs	Theoretically ≈ 1.4 MPa	High-pressure injection equipment

**Table 2:** On-site application of main water-blocking agents.

Type of Gas Reservoir	Gas Reservoir Characteristics	Common Types of Water Shutoff Agents	Field Application Effect
Fractured Gas Reservoir	Dual seepage of fractures and pores; concentrated high-permeability channels; severe heterogeneity	Polymer gels, prefabricated particle gels	Single-well water production decreased by 60–75%; gas production recovery rate reached 75–85%; the effect remained stable within two years
Sandstone Gas Reservoir	Fracture–pore composite seepage system; strong heterogeneity	Nano/polymer gels, polymer microspheres	Single-well water production decreased by 70–80%; gas production increased by 50–60%; the plugging layer operated stably under high temperature and high pressure for 18 months
Carbonate Gas Reservoir	Pore–fracture network formation; karst development; irregular pore structure	Thermosensitive gels, gas-wetting surface reversal agents	Single-well water production decreased by 65–70%; gas production recovery rate reached 80%; construction was simple with no near-wellbore plugging
Water-Driven and Horizontal Well Fracturing System	Significant differences in fracture width and permeability of multistage fracturing; complex fracture orientation	Multimolecular-weight polymers injected in layers, foam/microemulsion composites	Average water production of multistage fractures decreased by 70%; productivity was released evenly; bypassing of plugging was avoided
Condensate Gas Reservoir	High pressure differences drive condensable oil enrichment; mutual influence between pore-throat retention and gas-phase permeability reduction	Polymer microspheres and polymer bridging adsorbents	Single-well condensate oil recovery increased by 15–25%; water production rate decreased by approximately 50%; the condensate oil enrichment problem was effectively alleviated

**Table 3:** Application cases of polymer gas well water control.

Application Area	Reservoir Properties	Water Shutoff Agent	Application Effect
Verena Gas Reservoir, France	Sandstone; permeability (K) = 100–1000 mD; low salinity; temperature (T) = 30 °C	HPAM (with formaldehyde pretreatment fluid)	- Before treatment: gas relative permeability (Kᵣg) = 0.58; water relative permeability (Kᵣw) = 1.00 - After treatment: Kᵣg = 1.18; Kᵣw = 0.55
Wales Gas Well, Canada	Dolomit, ϕ = 9.0%, K = 200 mD, T = 113 °C	Activator and PAM	After treatment: gas production rate of 3 wells increased by 315%; water cut decreased by 65%
A Gas Reservoir in Northern Germany	Sandstone with shale interbeds; porosity (φ) = 12.7%; T = 120–130 °C; salinity = (25–33) × 10^4^ mg/L	VS, VA, and AM terpolymer	After treatment: water production decreased by 89 m^3^/d; gas production was restored
Western Germany Gas Field	Sandstone with clay interbeds; φ = 14.6–21.3%; K = 50–90 mD; T = 90 °C; salinity = 27 × 10^4^ mg/L	Partially sulfonated acrylamide terpolymer	- Before treatment: gas–water ratio (GWR) = 50,000 m^3^/m^3^ - After treatment: GWR > 250,000 m^3^/m^3^

**Table 4:** Gel gas well water control application cases.

Application Area	Reservoir Properties	Water Shutoff Agent	Application Effect
East Kalimantan Peciko Gas Field, Indonesia	Sandstone; porosity (φ) = 21.0%; permeability (K) = 500 mD; temperature (T) = 112 °C	Organic gel	Before treatment: gas production = 254,851.20 m^3^/d; water production = 15.89 m^3^/d
			After treatment: gas production = 252,019.52 m^3^/d; water production = 6.35 m^3^/d
East Kalimantan Gas Field, Indonesia	Sandstone; permeability (K) = 100–2000 mD; Temperature (T) = 115 °C	Aluminum gel	After treatment: gas production increased by 2.8 × 10^5^ m^3^/d
East High Island, Gulf of Mexico	——	Anionic PAM, dichromate gel	Before treatment: no gas production; water production = 95.38 m^3^/d
			After treatment: gas production = 53,801.92 m^3^/d; water production < 7.95 m^3^/d
Tainan Gas Field, Qinghai, China	Sandstone; porosity (φ) = 16.8–47.45%; salinity = (15–26) × 10^4^ mg/L	ZJ gel	Before treatment: gas production = 1200.00 m^3^/d; water production = 70.00 m^3^/d
			After treatment: gas production = 2700.00 m^3^/d; water production = 6.00 m^3^/d
India BaBassein Gas Field, Indiassein	Carbonate gas reservoir	Composite crosslinked gel plugging agent (hexamethylenetetramine and hydroquinone)	Before treatment: water production = 444.00 m^3^/d; no gas production
			After treatment: water production = 60.00 m^3^/d; gas production = 56,000.00 m^3^/d
A Gas Field in Oklahoma, USA	Carbonate rock; permeability (K) = 2–4 mD; temperature (T) = 149 °C; salinity = 22 × 10^4^ mg/L	PEI gel	After treatment: gas production increased by 7.7%; water cut decreased by 42%
Sukunka Oilfield, Britain	Fractured formation; temperature (T) = 77 °C	Chromium gel	After treatment: water–gas ratio (WGR) decreased by approximately 75%; gas production rate increased by 50%

**Table 5:** RPM well water control application cases.

Main Composition of Water Control System	Application Effect	Adaptability Analysis
HPAM-1 and HPAM-2	Water production reduced by 56 m^3^; gas production rate increased by 10%	Suitable for high water cut conditions
Sulfonated Acrylamide	Gas production increased fivefold; water production reduced by more than 80%	Better differential permeability reduction (DPR) effect at lower concentrations
Terpolymer STP	Water production reduced by 100%; gas production reduced by 37%	Suitable for high-temperature and high-salinity conditions
Brush-like RPM with Grafted MPEG Backbone	——	Suitable for high-temperature carbonate reservoirs
FG40-modified Nano-SiO_2_	Rock wettability transformed to super gas-wettability	Suitable for condensate gas reservoirs
Fluorocarbon Surfactant WA12	Gas production > 2 × 10^3^ m^3^/d	Temperature resistance up to 170 °C; salt resistance up to 7 × 10^4^ mg/L
Fluorosurfactant FG24	Gas phase permeability and gas production rate increased by more than 25%	Temperature resistance up to 120 °C

**Table 6:** Functional fluid gas well water control application cases.

Application Area	Reservoir Properties	Water Shutoff Agent	Application Effect
Sulige Gas Field, Shaanxi, China	Core porosity (φ) = 21.84%; permeability (K) = 1.1300 mD	Nanoemulsion of aminopolysiloxane and sodium fatty acid methyl ester sulfonate (MES)	After treatment: water relative permeability (Kᵣw) decreased by >60%; gas relative permeability (Kᵣg) showed minimal change
Tahe Gas Field (Condensate Gas Reservoir), Xinjiang, China	Temperature (T) = 140.0–150.0 °C; salinity = 20 × 10^4^ mg/L	Nano-active condensate oil	After treatment: water production rate was reduced to 1/9 of that before plugging
Tahe Gas Field (Condensate Gas Reservoir), Xinjiang, China	Medium-high permeability; temperature (T) > 120.0 °C; salinity > 15 × 10^4^ mg/L	Nanoparticle active oil	After treatment: single-well gas production increased by 7.0 × 10^4^ m^3^/d; water cut decreased by 75%
Yingmaili Gas Field (Condensate Gas Reservoir), Xinjiang, China	Temperature (T) = 106.7 °C	Water shutoff and gas-repellent emulsifier (blend of cetyltrimethylammonium chloride, octadecyltrimethylammonium chloride and modified oleic acid imidazoline)	After treatment: water shutoff rate reached 94.9%
Algiers Gas Field, Hungary	High permeability; strong heterogeneity	Silicone emulsion	After treatment: gas production increased by twofold
Kansas Gas Field, USA	——	Latex concentrate	Before treatment: water production = 11.1 m^3^/d After treatment: no water production; gas production recovered
Tunu Gas Field, Eastern Kalimantan, Indonesia	K = 0.9849 mD	Partially hydrolyzed aluminum oxide system	After treatment: water production decreased by 90%; gas production remained stable

**Table 7:** Challenges in researching gas reservoir water plugging materials. Definition of technology maturity used in Table 2 and Table 7. Initial stage = concept and laboratory proof-of-concept; development stage = validated in lab corefloods/microfluidics with reproducible metrics; demonstration stage = pilot/field trial with documented production response; industrial stage = repeatable commercial deployment with standardized QA/QC and supply chain.

Research Field	Limitations	Key Challenges	Domestic Technology Maturity
Basic Mechanism	Complexity of multifield coupling mechanisms	Processes such as adsorption, bridging, crosslinking, swelling, and dehydration overlap with each other, making it difficult to capture them simultaneously in a single experiment or model. It is challenging to quantify the conformation and interaction of polymer segments under high-salt, high-temperature, and complex pH conditions. Simultaneous micro- and nanoscale observation is required for bridging and retention; however, existing methods such as micro-CT and fluorescence microscopy cannot cover the full scale from nanometers to millimeters.	Preliminary microcharacterization and mechanical models have been established, but a systematic theory and multiscale verification platform have not been developed to date (in the initial stage).
New Material Design	Balance between temperature/salt resistance and migration ability	The coupling relationship between gelation reaction kinetics and migration distance is unclear. This may lead to two extremes: “premature solidification in the near-wellbore area” or “delayed gelation in the deep formation”. The mechanism of dynamic evolution of relative permeability in viscoelastic rheology and multiphase seepage is not fully understood, making it difficult to predict the “water-blocking and gas-preserving” diversion effect. The constitutive equation of the porous medium–polymer coupling simulation model lacks high-precision calibration, making it difficult to accurately extrapolate to on-site conditions.	Preliminary microcharacterization and mechanistic models have been established, but a systematic theory and multiscale verification platform have not been developed to date (in the initial stage).
On-Site Engineering Application	Strong formation heterogeneity	Fracture-pore heterogeneity leads to “bypassing plugging/skipping plugging”, making it difficult for the prepolymer solution to distribute evenly. On-site temperature, salinity, and pH fluctuate greatly, and the control of gelation timing is extremely sensitive. On-line monitoring data cannot quantify the microstructure of the gel network, making it difficult to optimize injection in real time. It is challenging to ensure the consistency of large-scale injection formulations, and on-site shearing and transportation may easily lead to premature crosslinking or degradation. Multistage zonal injection in horizontal wells requires precise zonal isolation and formulation switching, and downhole operations are complex. Construction costs are high, and risks are great, making it difficult to match economic evaluation with benefits. Long-term stability and the need for secondary opening coexist, and a unified standard for the design of gel breakers and reversible crosslinking bonds is lacking.	A few industrial demonstrations and pilot projects are ongoing, but the technical replicability, stability, and cost still require further optimization and verification (on-site demonstration stage).

## Data Availability

All the data used to support the findings of this study are available from the corresponding authors upon reasonable request.
